# Genetic Characterization in Familial Rotator Cuff Tear: An Exome Sequencing Study

**DOI:** 10.3390/biology11111565

**Published:** 2022-10-25

**Authors:** Alessia Azzarà, Laura Risi Ambrogioni, Ilaria Cassano, Carla Lintas, Umile Giuseppe Longo, Vincenzo Denaro, Fiorella Gurrieri

**Affiliations:** 1 Research Unit of Medical Genetics, Department of Medicine and Surgery, Università Campus Bio-Medico di Roma, Via Alvaro del Portillo, 21, 00128 Roma, Italy; 2 Operative Research Unit of Trauma and Orthopaedic Surgery, Fondazione Policlinico Universitario Campus Bio-Medico, Via Alvaro del Portillo, 200, 00128 Roma, Italy; 3 Research Unit of Trauma and Orthopaedic Surgery, Department of Medicine and Surgery, Università Campus Bio-Medico di Roma, Via Alvaro del Portillo, 21, 00128 Roma, Italy; 4 Operative Research Unit of Medical Genetics, Fondazione Policlinico Universitario Campus Bio-Medico, Via Alvaro del Portillo, 200, 00128 Roma, Italy

**Keywords:** rotator cuff, exome sequencing, genetics, gene, shoulder, tear

## Abstract

**Simple Summary:**

Rotator cuff tear is the most common musculoskeletal disorders in the working population. Family members of patients with rotator cuff disease have a significant higher risk of tendon tears than general population. The etiology is multifactorial, and its pathogenesis is not completely understood. It is expected that multiple gene variants contribute to disease susceptibility but, in some instances, a few main rare variants can be responsible for the clinical manifestations. We performed exome sequencing on a family (4 members) to identify rare gene variants predisposing to the development of rotator cuff tears. Rare variants in common between the two affected subjects were selected. Using the predictions in silico tools, the candidate variants were segregated by Sanger sequencing. Based on the risk of being damaging and on the potential role in rotator cuff etiopathogenesis, three candidate genes for rotator cuff tears were prioritized: COL23A1, EMILIN3 and HDAC10. These findings suggest that performing exome sequencing in other rotator cuff tears families will possibly lead to the identification of major candidate genes for the disease. The genes identified in the present study could be confirmed in a larger cohort of patients with rotator cuff tears, even if they have no family history.

**Abstract:**

Background: multiple gene variants seem to contribute to rotator cuff (RC) tear susceptibility. The aim of the study is to perform an exome sequencing analysis within a family to identify rare gene variants predisposing to the development of RC tear. Material and methods: the exome sequencing was conducted in a family consisting of four individuals, two healthy and the remaining ones with bilateral RC tears. Variants in common among the two affected subjects were selected, and those in common with the healthy subject and those with a frequency >1% were removed. The potential pathogenicity of the variants was investigated using the predictions of several in silico tools from VarSome. Results: the exome sequencing yielded approximately 600,000 variants per patient, subsequently filtered according to frequency <1% and absence of association with other diseases. Removing variants common with the healthy subject, 348 rare variants among 248 genes were identified. Based on the risk of damaging, three candidate genes for RC tear were found: COL23A1, EMILIN3, and HDAC10. Conclusion: this is the first whole-exome sequencing analysis within a family to explore genetic predisposition in RC tear. The results reveal the presence of common damaging variants among affected individuals in the COL23A1, EMILIN3, and HDAC10 genes.

## 1. Introduction

Rotator cuff (RC) tear is among the most common musculoskeletal disorders. In particular, RC tear is the third leading cause of musculoskeletal disease (16%), and its relevance is related to its high prevalence rate and disabling condition responsible for significantly direct and indirect socioeconomic costs. The incidence ranges from 5% to 39% and it increases in the elderly population, being approximately 6% and 30% in those under and over 60 years, respectively [1].

The etiology is multifactorial, and its pathogenesis is not completely understood. Some studies suggest that genetics plays a role in the pathogenesis of RC tear [2]. Family members of patients with RC tears have a significant higher risk of developing RC tear than the general population. Moreover, genetically susceptible patients manifest symptoms earlier and more often, with a relative risk of 1.44 for their siblings. Furthermore, over an observation period of five years, the progression of an RC lesion is more significant in siblings of an affected patient than in patients with no affected relatives [3,4].

Genome-wide association studies (GWAS) have made it possible to identify numerous disease-associated variants due to the large number of single nucleotide polymorphisms (SNPs) they analyze. GWAS represents a whole genome, or nearly whole genome, investigation of different individuals’ genes to determine the presence of gene variants in the sample population.

While GWAS can detect only gene variants common to many individuals in a larger population, whole-exome sequencing (WES) studies in addition focus on rare variants. The rationale behind the WES relies on the particular structure of our family, which suggests a mendelian transmission of susceptibility to RC tear (vertical transmission, bilaterality of lesions). Therefore, the objective was to identify new rare genetic variants of major impact segregating in affected members. Essentially, this is a “gene discovery study” [5].

In general, it is expected that multiple gene variants contribute to RC disease susceptibility but, in some instances, a few main rare variants can be responsible for the clinical manifestations [6,7]. For instance, Achilles tendinopathy has recently been associated with tenascin C and collagen type V-A polymorphisms, suggesting that variants in tissue structural proteins may play a role also in the pathogenesis of RC tears [8,9,10,11]. Other GWAS and case-control studies on RC tears have identified SNPs significantly associated with RC tears in *SASH1* (rs12527089), *SAP30BP* (rs820218), Cadherin-8, *DEFB1*, *FGFR1*, *FGFR3*, *ESRRB*, *FGF10*, *MMP*-*1*, *TNC*, and *FCRL3*. These genes are involved in apoptosis and cell adhesion. Contradictory results have been reported for MMP-3 [12,13,14,15,16,17,18,19]. GWAS have pointed to common variants more frequently detected in sporadic patients but, hitherto, no WES analysis has been conducted to identify rare gene variants with major pathogenetic role in familial cases.

The aim of this study is to perform a WES analysis on a RC tears family to identify rare gene variants predisposing to the development of RC tear.

## 2. Materials and Methods

This is an experimental observational study of exome sequencing. The study was conducted in accordance with the Declaration of Helsinki for Human Rights on a voluntary basis and was approved by the local ethics committee. All patients enrolled in the study signed informed consent.

### 2.1. Family

**Figure 1:**
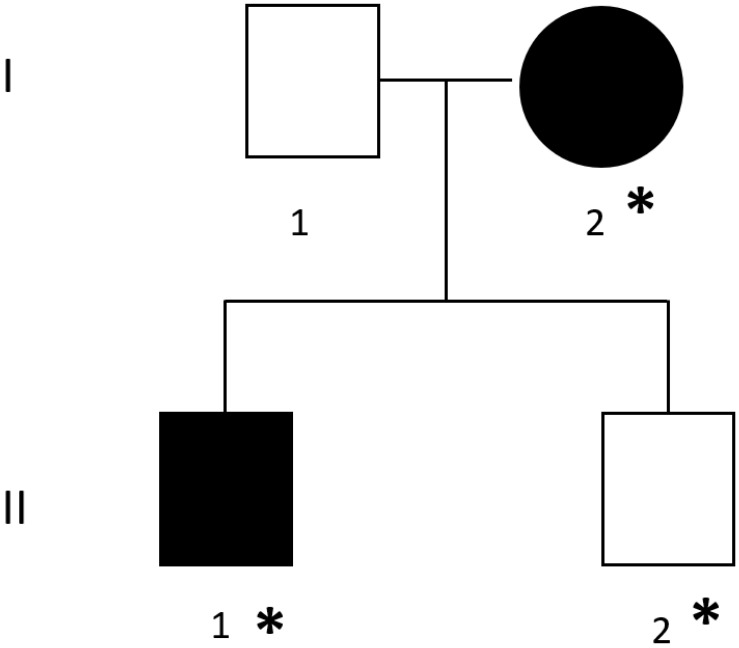
Pedigree of the family enrolled. The black symbols represent individuals with bilateral rotator cuff lesions. Asterisks stand for subjects who undergone Whole Exome Sequencing.

Figure 1 shows the pedigree of the family under investigation, in which patients I-2 and II-1 were diagnosed with bilateral full-thickness RC tears, confirmed by MRI. Each patient enrolled was given a thorough explanation of the study phases, the pathology examined, the purposes of the study, and the scientific significance of the collected data. None of the patients had a history of significant acute trauma to the involved shoulder and were of Italian origin.

The clinical overview of the patients involved in the present study is given below:

Subjects I-1 and II-2 had no shoulder pathologies during their lifetime. The absence of RC tear was assumed based on the complete absence of symptoms and the negative clinical examination.

Subjects I-2 and II-1 had bilateral full-thickness RC tears before age 55 without a history of trauma. A surgical procedure to repair the lesion was required.

DNA from blood samples of all patients was extracted.

### 2.2. Exome Analysis by Next Generation Sequencing

Exomes from patients I-2, II-1 and II-2 were sequenced in service at SRL (Pizzoli, L’Aquila AQ, Italy), requiring approximately 60X coverage on an Illumina sequencing platform. Paired-end sequencing was performed, and one file was obtained for each orientation. From paired-end sequencing, a pair of files in FASTQ format was obtained for each patient, which were mapped and filtered using the online platform UseGalaxy [20]. The analysis was customized by mapping reads to the human GRCh37/hg19 genome with Burrows-Wheeler Aligner tool [21], and duplicate reads were removed with the RmDup tool [22]. Variant calling was performed with FreeBayes tool. Variant Call Format (VCF) files from all three patients were merged with the bcf tools to create a multisample VCF file. We then re-filtered the entire file using regular expressions to identify only variants shared by the two affected subjects (I-2 and II-1), discarding variants shared with the healthy subject (II-2). The resulting single file, contains only the variants present in the affected subject, was annotated with wAnnovar and then filtered using Microsoft Excel functions. Specifically, the obtained variants were filtered for coding and splicing regions, excluding synonymous variants found in individuals of this family. To obtain information on the frequency of each variant, a search of the gnomAD database was performed. GnomAD (www.gnomad.broadinstitute.org 16 September 2022) is a resource developed with the intent of making sequencing data, both exomes and genomes, from a large number of large-scale sequencing projects available to the scientific community [23]. The amount of data in the GRCh37/hg19 dataset spans 125,748 exonic sequences and 15,708 genomic sequences from unrelated individuals sequenced as part of population genetics studies on specific diseases. Once the name of the gene of interest is entered, gnomAD, in addition to giving an idea of the coverage of the sequenced regions, provides a huge catalog of variants associated with that gene, the frequencies of which can be appreciated in different populations. Further shown are the structures of transcripts corresponding to the gene of interest, each with its own “ENST” abbreviation from the Ensembl database. The database provides the user with other information, including the ratio of the number of loss-of-function variants observed in a given gene over those expected, so as to return a measure of how tolerant a gene is to such variants. Variants with minor allele frequency (MAF) < 1% were prioritized. The potential pathogenicity of variants was investigated using predictions from several in silico tools via the VarSome website [24]. To prioritize, the literature was queried through VarElect software with the keywords “tendon”, “connective”, “rotator cuff”, and “tendinopathy”. This assessed whether and which genes obtained from filtering were most potentially associated with the pathogenesis of tendinopathies [25]. All genes and their variants were manually checked in the Binary Alignment Map (BAM) file, uploaded as a custom track on the UCSC genome browser, to assess their reliability with respect to coverage of the sequenced region.

### 2.3. Sanger Sequencing of Variants

Oligonucleotide primers flanking variants were designed using Primer3 application on the UCSC genome browser. Primer sequences will be provided upon request. Each amplicon was PCR amplified using the following standard cycles: 95 °C 5 min, 95 °C 30 s, 60 °C 30 s, 72 °C 15 s, for 34 cycles, final extension at 72 °C for 15 min. 2.5 microliters of each amplicon were purified with 0.5 microliters of a 1:1 mixture of Exonuclease III and Shrimp Alkaline Phosphatase at 37 °C for 15 min followed by heat inactivation at 80 °C. Cleaned up PCR product was sequenced using BigDye terminator v3.1 Cycle Sequencing Kit (Applied Biosystems, Foster City, CA, USA) in a final volume of 10 µL and run on a 3130 Genetic Analyzer (Applied Biosystems, Foster City, CA, USA). The electropherograms were analyzed by the Sequencing Analysis v5.2 software (Applied Biosystems, Foster City, CA, USA).

## 3. Results

**Figure 2:**
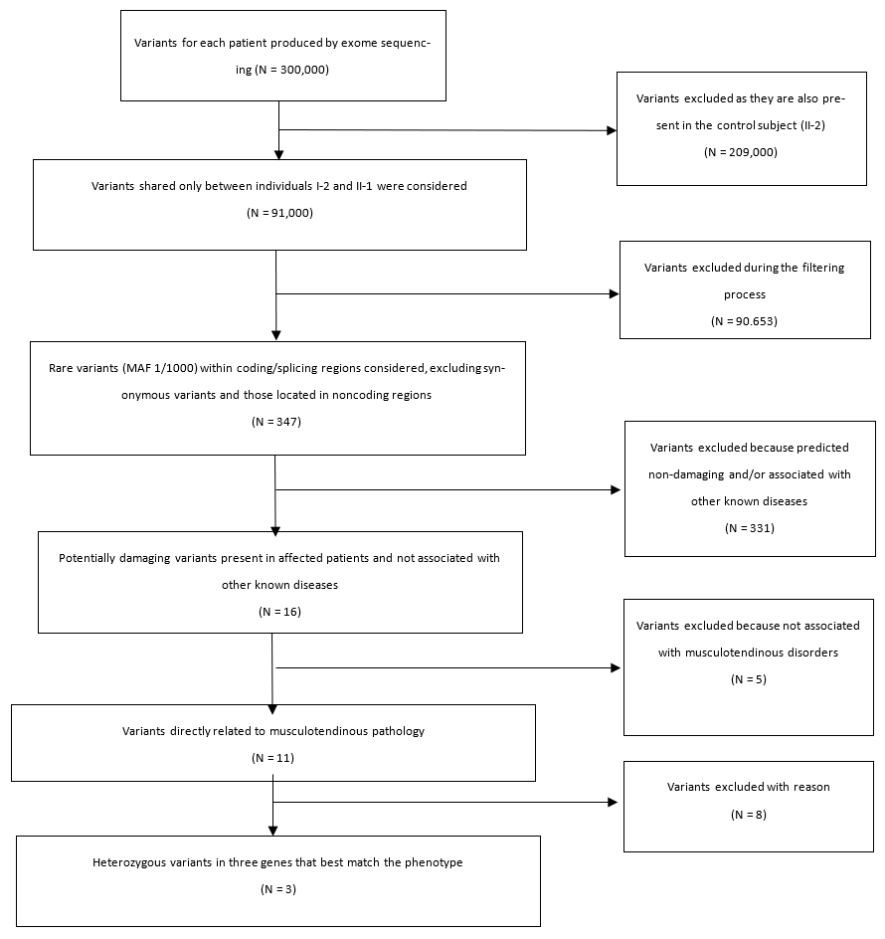
Flow-charts of the process of filtering and prioritization of variants obtained from WES.

We performed WES in a family of four individuals: father (I-1) and son (II-2) in apparent good health; mother (I-2) and son (II-1) suffering from bilateral RC tears. We followed different filtering steps (summarized in Figure 2) in order to identify candidate genes. All bioinformatic analyses were carried out following best practices recommendations [26,27].

Exome sequencing yielded approximately 300,000 variants for each patient. After merge, the variants in common with the healthy subject (II-2) were filtered out, obtaining 91,000 variants shared between I-2 and II-1 individuals. Subsequently only rare variants (MAF 1/1000) within the coding/splicing regions were filtered in, excluding synonymous variant and those located in non-coding regions. We obtained 347 rare variants present only in the affected mother (I-2) and son (II-1). From these variants, we collected 16 variants in 16 genes with read depth cut-off >20 in the BAM file, predicted as damaging with in silico predictive tools on VarSome website and not associated with other known diseases (Table S1).

**Figure 3:**
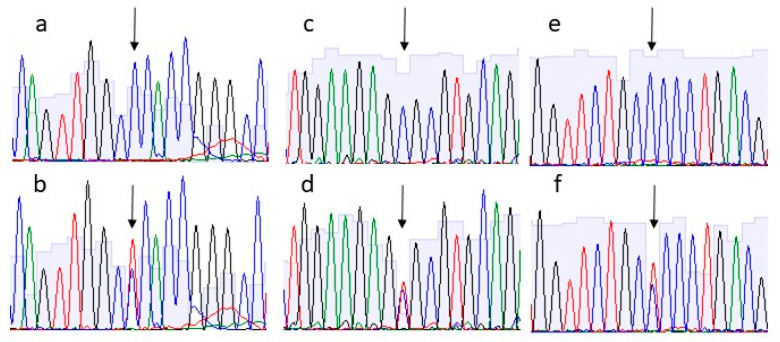
Electropherograms of the variants tested. Panel (**a**,**c**,**d**) showing the wiltype variant of COL23A1, EMILIN3 and HDAC10 genes, respectively, present in the two healthy members of the family. Panel (**b**,**d,e**) showing the heterozygous variant in COL23A1, EMILIN3 and HDAC10 genes, respectively, identified in the two subjects with RC tears. Arrows indicate the site of substitution.

To prioritize them, we used VarElect software with the keywords (“tendon”, “connective”, “rotator cuff”, and “tendinopathy”) to assess whether and which of the 16 genes obtained were most likely associated with the pathogenesis of tendon diseases. Among them, only 11 genes resulted directly related to the match keywords. At the end, we selected three heterozygous variants on basis the strength of the connection between the genes and the queried phenotypes to be further pursued by Sanger sequencing: c.1372C>T p.(Pro458Ser) in *COL23A1* gene, c.1417C>T p.(Arg473Cys) in *EMILIN3* gene and c.1277C>T p.(Pro426Leu) in *HDAC10* gene. All these variants were shared by the two affected subjects but not by the healthy family members (Figure 3).

## 4. Discussion

This is the first exome sequencing study to explore the major rare variants that contribute to genetic predisposition to RC tears. Our results provide preliminary evidence on the possible etiopathogenetic role of three genes (*COL23A1*, *EMILIN3* and *HDAC10*) and put the bases for designing further studies to confirm and broaden the available results. Despite several genetic loci have been associated with RC tears in previous GWAS, very few of these have been replicated to confirm their actual etiopathogenetic role. Since polygenic inheritance is suspected to underlie the disorder, due to genetic heterogeneity the variants identified in some studies may not be present in all affected patients. We designed the present study to obtain as relevant results as possible by reducing this heterogeneity. For this reason, we decided not to conduct the sequencing analysis in a cohort of affected patients but to proceed through a family-based approach. In this way, finding genetic variants segregating in affected family members increases the probability that those genes give a major contribution to the disease phenotype. In addition, to reduce the effects of intra-family heterogeneity, we excluded families whose individuals were affected by other shoulder diseases such as osteoarthritis or frozen shoulder. Part of our strategy to increase the homogeneity of the sample was to select a family in which RC tear occurred bilaterally in at least one individual without a history of trauma. This exome sequencing study identified 16 damaging and rare variants on 16 genes that segregate with the phenotype of affected family members and are absent in the healthy subject II-2. Among these 16 identified variants, we conducted prioritization by phenotypic matching, and three genes were found to have a higher correlation with disease. However, we cannot rule out the possibility that the other variants found in other genes may still be related to RC tears. Therefore, further studies on other families with RC tears are needed to evaluate their possible role.

Segregation analysis showed that the missense variants in *COL23A1*, *EMILIN3* and *HDAC10* genes were not present in the healthy father.

*COL23A1* (Collagen Type XXIII Alpha 1 Chain) is a member of the transmembrane collagens, a subfamily of the collagens that contain a single pass hydrophobic transmembrane domain. The protein is expressed in the epidermis and other epithelia (tongue, gut, and lung) but also in the brain and kidney [28]. In prostate, collagen XXIII expression was shown to correlate with tumor progression, such as in non-small cell lung cancers [29,30]. Although this protein has not been so far associated with musculoskeletal disorders, the high conservation of identical *COL23A1* nucleotide sequences in other collagen genes could suggests a possible role in tissue stability. Future studies should clarify its presence in musculoskeletal tissue.

*EMILIN3* (Elastin Microfibril Interfacer 3) gene encode for a protein related to extracellular matrix organization and elastic fiber formation. In situ hybridization of day-9.5 mouse embryos detected expression during skeletal development, particularly at sites of cartilage and bone formation [31]. Study in mesenchymal stem cells identified EMILIN3 protein in osteoblastic differentiation and immunohistochemical analysis of day-13.5 mouse embryos found EMILIN3 expressed on the perichondrium of murine limbs and vertebral bodies [32]. Further studies are needed to validate its expression on the human perichondrium to understand better the molecular role of the protein.

*HDAC10* (Histone Deacetylase 10) gene encodes a member of histone deacetylase family, which deacetylate lysine residues on the N-terminal part of the core histones. Histone deacetylation, involved in chromatin structure, plays an important role in transcriptional regulation, cell cycle progression, and developmental events. An aberrant expression of histone deacetylases (HDACs) is associated with carcinogenesis [33]. SNPs in MMPs have also been closely associated with RC tears [12,13,14,15,16,17,18,19,34]. In addition, *HDAC10* is important in chronic inflammation, and inflammatory responses affect synovium-derived mesenchymal stem cell (SMSC) function in temporomandibular joint repair [35]. In fact, SMSCs play a major role in musculoskeletal regeneration, particularly for reconstructions of cartilage, bones, tendons, and muscles [36]. Analysis of the expression levels of these genes at RNA and protein level in diseased RC tear tissues could clarify their role in this condition. Our results will be strengthened by finding variants in these same genes in other WES of RC tears families/cases. New functions of the proteins encoded by *COL23A1*, *EMILIN3* and *HDAC10* genes might be established.

Considering the genetic heterogeneity of RC tear, it is not surprising that we ended up with different genes. The finding of multiple genes in our study is in agreement with the data available in the literature and confirms that RC tear is a polygenic disease. Our analysis started by searching for genes reported in the literature as potentially contributing to the development of RC tear. We thoroughly analysed in our WES all the genes previously reported in GWAS studies but found no alteration in our family. Although this result may appear contradictory, awareness of the inherent differences between GWAS and WES studies makes it possible to understand the reason behind this finding. In the present WES study, we only searched rare variants of high impact in a single family, whereas GWAS results come from the search of common variants of lower impact in large cohorts, thus picking more genes. The most reasonable interpretation of this apparent inconsistency relies on the complex genetic architecture of multifactorial diseases, which include RC tear: in fact it usually happens that the majority of affected individuals carry multiple low impact variants in different genes which add up to overcome a threshold (polygenic disease model), whereas only a minority of patients have the same phenotype due to one single variant of major impact in one or very few genes (mono- or oligogenic architecture). Therefore, the finding of rare variants potentially damaging to the development of RC tear is not in conflict with the genetic mutations reported in the literature. Our study demonstrates the advantages of a WES study in identifying rare variants and understanding the heritability of the disease.

Finally, the results of this study show how the study of variants is critical to understanding the molecular mechanisms of RC tears. In particular, variants in the COL23A1, EMILIN3, and HDAC10 genes seem the most interesting for their role in musculoskeletal tissue. Further studies are expected to comprehend how these mutations influence the phenotype. Furthermore, considering the cost-effectiveness of WES studies in identifying rare variants, future exome sequencing analyses should be conducted to confirm and extend this list of candidate variants for RC tear development. These segregating variants will be the starting point for identifying variants in the same genes in a larger cohort of patients with RC tears, even without a family history of the disease. The comprehension of disease pathogenesis will enable early molecular diagnosis and personalized treatment choices to lower healthcare costs and improve patients’ quality of life thereafter. Moreover, genetic predisposition as a risk factor for RC tears raises the possibility of looking for mutations in structural proteins in other tendons, clarifying the relationships between RC tears and other tendinopathies.

## 5. Conclusions

This is the first whole-exome sequencing analysis conducted within a family to assess the genetic predisposition of RC tear. The results show a list of candidate genes for the disease. Among 16 genes considered to be damaging, rare, and present only in affected individuals, variants in the *COL23A1*, *EMILIN3*, and *HDAC10* genes appear to be the most interesting for their role in musculoskeletal tissue. Performing the same study on other families could increase the evidence for the potential role of these candidate genes in the disease.

## Data Availability

Not applicable.

## Supplementary Materials

The following supporting information can be downloaded at: https://www.mdpi.com/article/10.3390/biology11111565/s1, Table S1: Potentially damaging variants present in affected patients, but not in healthy ones.
