# Supplementary material for: Genetic Characterization in Familial Rotator Cuff Tear: An Exome Sequencing Study
Source: Biology (Basel). 2022 Oct 25;11(11):1565. doi: 10.3390/biology11111565 (PMC9687989; doi:10.3390/biology11111565)
Supplement: Supplementary file 1 [file biology-11-01565-s001.zip › biology-1948480-supplementary.pdf]

Table S1 - Potentially damaging variants present in affected patients, but not in the healthy ones.

Gene	Chr	Position	DNA & AA change	gnomAD MAF (%)	*In silico* prediction tools	CADD score
LINGO4	1	151774279	NM_001004432:c.902G>A:p.(Arg301Gln)	0,026	10/18 damaging	28,5
RNF149	2	101911601	NM_173647:c.503G>T:p.(Gly168Val)	0,001	9/12 damaging	29,8
C2orf40	2	106690387	NM_032411:exon3:c.C173T:p.A58V	0,0004	9/11 damaging	24,6
DBI	2	120125869	NM_001079862:c.115G>A:p.(Asp39Asn)	0,466	.	24,4
COL23A1	5	177673296	NM_173465:c.C1372C>T:p.(Pro458Ser)	0,062	8/12 damaging	23,9
JARID2	6	15496817	NM_004973:c.1361A>C:p.(Gln454Pro)	.	8/12 damaging	22,7
MDC1	6	30681083	NM_014641:c.618_636del:p.(Gly207ProfsTer3)	0,21	1/1 damaging	24
UPK2	11	118827665	NM_006760:c.149C>A:p.(Pro50His)	0,049	12/20 damaging	26,3
KIFC3	16	57803855	NM_005550:c.952G>A:p.(Glu318Lys)	0,024	10/12 damaging	27,7
ABCA9	17	66981048	NM_080283:c.4357C>A:p.(Pro1453Thr)	0,41	8/11 damaging	24
GIPR	19	46181024	NM_000164:c.911T>G:p.(Leu304Arg)	0,016	8/12 damaging	28,2
EHD2	19	48239657	NM_014601:c.947A>G:p.(Lys316Arg)	0,011	14/19 damaging	22,8
EMILIN3	20	39990792	NM_052846:c.1417C>T:p.(Arg473Cys)	0,006	11/19 damaging	26,3
PREX1	20	47307634	NM_020820:c.1037C>T:p.(Ala346Val)	0,001	10/19 damaging	28,1
GATD3	21	45560145	NM_004649:c.442G>A:p.(Val148Met)	0,95	6/9 damaging	23,5
HDAC10	22	50686379	NM_032019:c.1277C>T:p.(Pro426Leu)	.	.	24

Chr = Chromosome
AA change = Aminoacid change
GnomAD MAF = Minor allele frequency of the identified variant in the population reported by the GnomAD database
CADD score = deleteriousness score of single nucleotide variants and insertion/deletion variants in the human genome
